# Gender Differences in Cognitive Impairment among Rural Elderly in China

**DOI:** 10.3390/ijerph17103724

**Published:** 2020-05-25

**Authors:** Jing Wang, Lily Dongxia Xiao, Kai Wang, Yan Luo, Xiaomei Li

**Affiliations:** 1 Faculty of Nursing, Health Science Center, Xi’an Jiaotong University, 76# Yanta West Road, Xi’an 710061, China; luoyan0904@xjtu.edu.cn (Y.L.); roselee@xjtu.edu.cn (X.L.); 2 College of Nursing and Health Sciences, Flinders University, Sturt Road, Bedford Park, Adelaide, SA 5001, Australia; lily.xiao@flinders.edu.au; 3 Xiang Ya Nursing School, Central South University, 172# Tongzipo Road, Changsha 410013, China; 4 Department of Epidemiology and Biostatistics, School of Public Health, Tongji Medical College, Huazhong University of Science and Technology, 13# HangKong Road, Wuhan 430030, China; kay_wang@hust.edu.cn

**Keywords:** cognitive impairment, epidemiology, risk factors, gender difference, China

## Abstract

Elders in rural areas of developing countries are particularly vulnerable to cognitive impairment and gender disparities are notable. Yet, evidence related to gender differences in cognitive impairment in these settings is scarce. This study examined gender differences in cognitive impairment among elderly people aged 65 and above in rural China. A multi-site cross-sectional survey was conducted. In total, 550 males and 700 females were recruited. The Chinese version of the Mini-Mental Status Examination was applied for cognitive impairment screening. Demographic information, lifestyle factors, psychosocial factors, and health-related information were assessed. The prevalence of cognitive impairment was 40.0%in males and 45.1% in females, respectively. Females showed significant higher prevalence after age 75 (62.7% vs. 45.4%, *p* < 0.005). Older age, hearing impairment, and activities of daily living dependence were common factors associated with cognitive impairment in both females and males. For males, living in a neighborhood with poor social interactions and diabetes were risk factors, while tea consumption was a protective factor. For females, vision impairment and illiteracy were additional risk factors. Individualized interventions and standardized measurements of cognitive function should be developed to suit older populations living in rural and less-developed areas, through collective efforts involving all stakeholders and multidisciplinary teamwork.

## 1. Introduction

Dementia is the leading cause of disability among people aged 65 years and over across the world. Dementia is identified as a public health priority by the World Health Organization and they are calling for global and national responses to address the impact of dementia as an increasing threat to global health [1]. Estimates indicate that 50 million people are living with dementia around the world, and there will be a new case every three seconds, leading to about 152 million people living with dementia by 2050 [2]. With the extension of life expectancy, the largest increases in dementia cases are predicted to occur in low- and middle-income countries(LMICs) which currently have undeveloped dementia care systems and few research studies conducted there [2]. This study addresses the gap in research by exploring gender differences in the prevalence of cognitive impairment in rural areas of LMICs, using China as a case, and seeks to identify unique risk factors for women and men, in order to provide evidence for individualized preventive interventions and policy development in dementia care.

Considering the current absence of curative treatments and the huge burden caused by dementia, growing interest and focus are on risk reduction and disease prevention. This requires the identification of modifiable risk factors as a first step in order to develop effective preventive strategies. There is sufficient evidence from observational studies conducted in high-income countries (HICs) that supports the association between several modifiable risk factors and a reduced risk for cognitive impairment and dementia [3,4]. Evidence from the first large multi-dimensional lifestyle trial, the Finish Geriatric Intervention Study to Prevent Cognitive Impairment and Disability (FINGER), shows that at least one third of dementia cases are associated with modifiable factors (i.e., physical activity, diet, diabetes, obesity, and cognitive stimulation), although this figure is estimated to be more like 50% in reality [2,5].

Recognized risk factors for cognitive impairment can be categorized into demographic risk factors (i.e., advanced age), genetic factors (for example, APOE gene), cardiovascular disease (such as hypertension, diabetes, dyslipidaemia, and obesity), psychiatric risk factors (i.e., depression, psychological distress, and sleep disorders), and lifestyle (for example, smoking, drinking) [4,6]. High physical activity, cognitive stimulation, education attainment, and healthy dietary pattern such as Mediterranean diet are identified as protective factors for cognitive impairment [4,5,6]. It is estimated that a third of late life cognitive impairment might be attributable to seven common modifiable risk factors: diabetes mellitus, midlife hypertension, midlife obesity, physical inactivity, depression, smoking, and low education [4,5]. A reduction in the prevalence of dementia due to these factors by 10%–20% per decade could reduce the prevalence of Alzheimer’s disease by up to 50% in 2050 [2,3]. Consistent with these risk factors for dementia, the incidence of dementia in HICs has been declining for the past ten years, possibly because of improvements in cardiovascular health, increased educational attainment, improved lifestyle generally, and reduced incidence of stroke [5,7].

Inconsistent findings are reported with regard to gender differences in dementia prevalence, with more studies indicating higher prevalence among females than in males [8,9]. Some studies indicate that gender difference varies according to the type of dementia, with higher prevalence of Alzheimer’s disease in females and more vascular dementia in males [10,11]. Males show steeper declines than females in areas of speed, integration, and visuospatial ability, while steeper declines in delayed recall and executive function are found in females [11,12]. Gender differences in socio-economic status (i.e., education, income, occupation, family roles), psychosocial factors (i.e., coping, social engagement), cardiovascular and metabolic diseases, sex hormone changes in midlife, genetics, and cognitive reserves, might contribute to prevalence variations [10,13]. Better understanding of gender differences in the prevalence and risk factors of cognitive impairment is needed for personalized intervention development to better mitigate risk factors.

Existing data on gender differences in dementia prevalence are predominantly gathered from HICs. However, predictors of dementia are complex and vary in different settings depending on race, ethnicity, culture, lifestyle, genetics, economic, and social context [7,14]. These factors might jointly contribute to prevalence differences in dementia between urban and rural areas, and between LMICs and HICs. Despite the declining incidence of dementia in western countries, the opposite trend is reported in Asian countries, including China, which are showing increasing incidence and prevalence of dementia [8,15]. Almost half of the worldwide prevalence and incidence of dementia resides in Asia, but two thirds of Asian countries are LMICs, that are not economically and socially prepared to cope with the consequences [2,15]. The limited dementia services in these countries are usually only available to metropolitan residents [8,16]. Moreover, many developing countries are also undergoing an epidemiological transition, with dramatic increases in the prevalence of cardiovascular diseases that are correlated with dementia incidents [15].

The prevalence rate of dementia in rural areas is substantially higher than in urban areas, and the risk factors for dementia differ between rural and urban populations [7,16]. Yet, prevalence of, and risk factors for, cognitive impairment in rural regions have not been adequately addressed in Asian countries. Given low educational attainment and health literacy, very limited access to social protection and health services, and much fewer studies conducted in rural populations [8,17], it is of paramount importance to identify the prevalence and risk factors of cognitive impairment among rural populations in developing countries, to inform policy, interventions, and service development for dementia prevention. The aim of this multi-site cross-sectional study was to explore gender differences in the prevalence of and risk factors for cognitive impairment among the rural elderly aged 65 years and over in China. This is part of a longitudinal study using the baseline data. The rest of the paper is divided as follows: materials and methods, results, discussion, and conclusions. The next section outlines the materials and methods used in this study to address the research aim.

## 2. Materials and Methods

### 2.1. Ethical Considerations

This study was conducted in accordance with the Declaration of Helsinki, and the protocol was approved by the Ethics Committee of Health Science Center, Xi’an Jiaotong University, as well as the local hospitals (Project identification code: 2016-221) that facilitated the survey. All participants were provided detailed information about this study, including potential risks and benefits, and their rights. All participants gave their informed consent for inclusion before they participated in the study. Permission from participants and local hospitals was gained to review health records. Local hospitals have an electronic health record system for all residents aged 65 years and over in their designated area, which is updated annually based on the free annual health assessment results.

### 2.2. Setting and Participants

**Figure 1:**
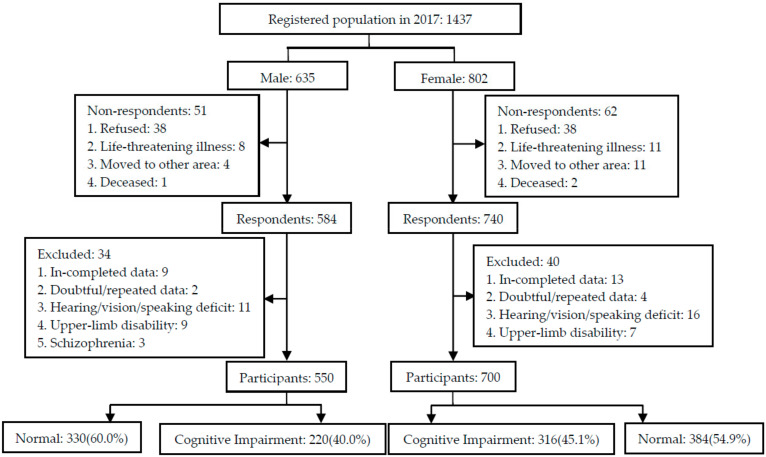
Study flow chart.

Participant recruitment and data collection were completed from June to September, 2017. Ten villages were randomly selected from one province located in the north of China. All residents who were aged 65 years or older, listed in the registry office census, and had lived in the selected villages for the past 5 years or more before the survey, were invited to participate in this study. Physicians in the local village clinics helped with participant recruitment. Residents who declined to participate, suffered from a life-threatening illness, moved to other areas, or passed away were excluded. In total, 1437 residents (635 males and 802 females) were drawn from these 10 villages, of which 1324 (584 males and 740 females) responded to the survey. Respondents who provided in-completed data, doubtful/repeated data, or were unable to complete the assessment due to hearing/vision/speaking deficit, upper-limb disability or schizophrenia were excluded. Finally, 1250 participants, including 550 males and 700 females, were recruited from the villages. The study flow chart is presented in Figure 1.

### 2.3. Data Collection

Data collection was conducted via the free annual health assessment program for the elderly aged 65 years and over, funded by the Chinese Government. Physicians working in village clinics helped invite eligible residents based on the census register. After their annual health assessment, residents were invited to participate in the study and informed consent was obtained directly from each participant or a family caregiver. Face-to-face individual interviews lasting about 30 min were conducted to collect data in local village clinics by the investigators, who were researchers and health professionals with at least ten years of experience working in public health recruited from the local township hospitals.

A standardized two-day training program about data collection procedures was provided to all investigators, and a pilot survey with 50 rural elders was carried out to guarantee the measurements. A retraining course lasting around two hours was conducted after the pilot survey, and refresher training was provided as needed during the investigation process. Modifications were made to the measurements, especially the demographic information sheet, according to findings from the pilot survey. The researchers checked data before the end of each survey day, and logical errors or missing information were handled the following day by contacting the village doctor or the participant directly. Data collection included demographic information, cognitive function, lifestyle, psychosocial factors, and health-related factors. Measurements are described below.

#### 2.3.1. Demographic Information

Participants’ demographic information was collected through a demographic information sheet, including age, gender, marital status, education, family income, and living situation.

#### 2.3.2. Cognitive Function

The Chinese version of the Mini-Mental Status Examination (MMSE) was applied to assess participants’ cognitive function, including orientation to time and place, registration, attention and calculation, recall, language, and visual construction [18]. The MMSE is widely used globally for cognitive impairment assessment, with excellent sensitivity and specificity. The MMSE consists of 30 items, with each item rated as 0 or 1 according to the interviewee’s response, yielding a total maximum score of 30. Participants with higher scores indicate a better cognitive function. Cut-off points, based on the education background of the interviewee, were ≤17 for illiteracy, 20 for primary school, and 24 for secondary school [19]. The Chinese MMSE showed a Cronbach’s alpha coefficient of 0.722 in this study.

#### 2.3.3. Lifestyle Factors

Lifestyle factors were investigated via a self-report approach. A lifestyle information sheet was developed based on a literature review and expert panel review. Revisions to the information sheet were made according to expert feedback and findings from the pilot study. Information collected included regular house/farm work, smoking, alcohol consumption, and habitual tea consumption.

#### 2.3.4. Psychosocial Factors

A self-reporting approach was used to collect information about sleep quality, memory complaints, and social interactions with others in the neighborhood. Through self-reporting, participants rated their social interactions as poor, satisfactory or good.

Coping style was assessed using the 20-item Simplified Coping Style Questionnaire (SCSQ) [20]. The SCSQ includes two dimensions of coping style, positive coping with 12 items and negative coping with 8 items. Each item was rated on a 4-point Likert scale, ranging from 0 (never use) to 3 (often use). The total score was the mean difference of positive coping score and negative coping score, with a positive number indicating a positive coping style, while a negative number indicates a negative coping style.

#### 2.3.5. Health-Related Factors

The health-related factors investigated included cardiovascular risk factors and other chronic conditions. Four approaches, including health records, self-reports, physical examinations, and assessment scale, were employed to collect data about health-related risk factors. Health records were reviewed to collect comprehensive information about health-related factors, including hypertension, type 2 diabetes, cerebrovascular disease, heart disease, hyperlipidemia, and renal disease. Supplementary information not included in electronic health records, such as hearing impairment, and traumatic brain injuries, was sought through self-report questions. Height, weight, waistline, and vision were measured by trained health professionals using the same instrument. Body mass index (BMI) was then calculated and categorized into one of three groups: underweight, normal weight and overweight.

The Activities of Daily Living Scale (ADLS) was applied to assess both physical activities of daily living (ADL) and instrumental ADL [21]. The ADLS is a 14-item scale, with each item rated on a 4-point Likert scale, ranging from 1 (totally independent) to 4 (totally dependent), yielding a maximum total score of 56. Higher scores indicate higher dependence. A total score of 22 points or above, or more than one item rated as 3 points or more, was identified as ADL dependence. The physical ADL subscale includes 6 items with a total score of 6 points, indicating dependence in physical ADL. The IADL subscale consists of 8 items with a total score of 8 points, indicating IADL dependence. The Cronbach’s alpha coefficients of ADLS and two subscales in this study were 0.814~0.894.

### 2.4. Data Analysis

Data were entered into EpiData 3.1 (The Epidata Association, Odense, Denmark) on two separate occasions by two researchers and exported to SPSS 22.0 (IBM, Chicago, USA) for statistical analyses. Descriptive statistics were expressed as means and standard deviations for continuous variables, whereas frequencies were calculated for categorical variables. Characteristics of males and females were compared by independent-samples *t* test and χ^2^test.Estimates of the prevalence of cognitive impairment in females and males were calculated separately.χ^2^testwasused to examine prevalence differences among subgroups. Univariate logistic regression was performed to select possible risk factors for cognitive impairment among males and females. Multivariate logistic regression models with backward method were further applied to ascertain the predictors for cognitive impairment among males and females, considering the main effects of socio-demographics, lifestyle, psychological factors, and health-related factors. Variables significantly associated with cognitive impairment in univariate analysis and reported by previous studies were entered as independent variables for multivariate regression analyses. A *p* value less than 0.05 in two-tailed testing was considered statistically significant.

## 3. Results

A total of 1250 residents (550 males and 700 females) participated in the study and provided complete data. There were no significant differences in the participation rate for males and females (86.6% vs. 87.3%, *p* > 0.05).

### 3.1. Demographic, Lifestyle, and Psychosocial Information

Major characteristics of all participants are presented in Table 1. The mean age of both males and females was around 73 years old, with a range of 65~89 years for males and 65~92 years for females. No significant age differences were found between male and female participants (*p* > 0.05). Compared with male participants, more female participants were divorced/widowed (40.4% vs. 19.3%), illiterate (15.4% vs. 4.9%), engaged in physical activities such as house/farm work (80.4% vs. 58.7%), and complained of memory decline (86.3% vs. 78.2%). More than half of males had a secondary school education or above, while the proportion of females completing secondary school was only 28.6% (*p* < 0.001). Up to 80% of female participants had an income lower than the poverty line, which was much higher than the percentage of males (45.3%, *p* < 0.001). Living with a spouse was more common for males than females (44.2% vs. 30.3%, *p* < 0.001), who were more likely to live with an extended family of three or more generations (47.7% vs. 41.5%, *p* < 0.001). Compared with female participants, males were more likely to smoke, consume alcohol, and drink tea (*p* < 0.005). No significant difference between males and females was found in neighborhood relations or coping strategy.

### 3.2. Health-Related Information

The health-related information of participants is shown in Table 2. Females had a higher proportion of high waistline than males (65.1% vs. 51.8%, *p* < 0.005). The proportions of females with hypertension, diabetes, hyperlipidemia, and two or more chronic conditions were significantly higher than males, with exceptions of renal disease and traumatic brain injury (*p* < 0.05). No significant differences were found in hearing deficit, vision impairment, cerebrovascular disease, or heart disease between male and female participants. Although there was not a significant difference in overall ADL dependence, physical ADL dependence and IADL dependence were more common in females than in males (*p* < 0.05).

### 3.3. Cognitive Impairment inMales and Females

The prevalence of cognitive impairment was 40% (95% CI, 35.9–44.1) in males and 45.1% (95% CI, 41.4–48.8) in females (*p* > 0.05). The mean MMSE total score of female elders was significantly lower than males (21.26 vs. 23.12, *p* < 0.001). For both males and females, prevalence increased with age, reaching 57.1% in males and 71.0% in females aged 80 years and over. Prevalence was higher in females than in males after the age of 75 years (*p* < 0.01). Illiterate individuals showed highest prevalence, with significant higher prevalence in females than in males (74.1% vs. 48.1%, *p* < 0.01). No significant differences in prevalence were found between males and females with regard to subgroups stratified by marital status, income and living situation. Detailed information is shown in Table 3.

### 3.4. Univariate Analysis of Factors Associated withCognitive Impairment

Univariate analysis of demographic, lifestyle and psychosocial factors for cognitive impairment is presented in Table 4. For males, older age, having the government basic living allowance as the only income source, and poor social interactions in the neighborhood were associated with higher risk of cognitive impairment, while regular house/farm work, moderate alcohol consumption, and habitual tea consumption were associated with lower risk (*p* < 0.05). For females, older age, divorced/widowed status, illiteracy, having the government basic living allowance as the only income source, poor sleep quality, and memory complaints were found to be correlated with higher risk of cognitive impairment, while regular house/farm work, living with a spouse, and positive coping strategies were correlated with lower risk (*p* < 0.05).

The univariate analysis of health-related factors for cognitive impairment is presented in Table 5. For both male and female participants, poor hearing, weak vision, more chronic diseases, and ADL dependence, including physical ADL and IADL dependence, were significantly related to higher risk of cognitive impairment (*p* < 0.05). Higher BMI was relevant to lower risk of cognitive impairment among female participants (*p* < 0.05). ADL dependence (OR 3.551, 95% CI1.702~7.409) and weak vision (OR 3.867, 95% CI 2.175~6.872) showed strongest correlation with cognitive impairment in males and females, respectively.

### 3.5. Multivariate Analysis of Factors Associated withCognitive Impairment

Multivariate analysis found that older age and hearing impairment were common factors associated with higher risk of cognitive impairment in both females and males. The risk of cognitive impairment was greater in males living in neighborhoods with poor social interactions with others (OR, 4.860; 95% CI, 1.424–16.583), diabetes (OR, 2.344; 95% CI, 1.083–5.076) or ADL dependence (OR, 3.267; 95% CI, 1.459–7.314), whereas the risk was greater among females with weak vision (OR, 2.024; 95% CI, 1.051–3.897), or physical ADL dependence (OR, 1.613; 95% CI, 1.121–2.322). The risk for cognitive impairment was lower for males who consumed tea regularly (OR, 0.656; 95% CI 0.431–0.999), whereas it was lower for females who had completed formal school education (OR, 0.352; 95% CI 0.199–0.623). The overall models were statistically significant for both males and females (*p* < 0.001). Multivariate analysis results are presented in Table 6.

## 4. Discussion

This study explored the prevalence of and factors associated with cognitive impairment in rural residents aged 65 years and older, and gender differences were compared. Such results have rarely been reported in previous studies, especially forrural regions of China. Factors analyzed in this study covered socio-demographic factors, lifestyle factors, psychosocial factors, cardiovascular factors, and other health-related factors that have been identified in populations from Western countries and urban regions [3,22]. Some of these factors had not been previously explored in rural populations from Asian countries. Furthermore, data on cardiovascular diseases and other chronic conditions analyzed in this study were collected from hospital health records rather than from self-reported questions. This study addresses the gap in research regarding prevalence estimates and risk factors of cognitive impairment in rural areas [2,15].Gender differences identified in this study could help inform individualized interventions and service developments in dementia prevention.

The prevalence of cognitive impairment in males and females was 40.0% and 45.1%, respectively. These prevalence figures are significantly higher than the rates reported in previous study, which had a focus on residents in urban areas and reported rates of cognitive impairment at 32.9% and 15.7% for females and males aged 80 years and over, respectively [17].The relatively poor socioeconomic status of residents in rural areas, increased life expectancy, lifestyle changes over the past decade, and a much higher participation rate in this study may contribute to the higher prevalence rates identified in this study.

Although the overall prevalence was found to be higher in women than in men, these differences did not reach statistical significance. Before the age of 75 years old, the prevalence rate is similar between female and males, while a significant higher prevalence was found after age 75 years in women. Gender differences in cognitive impairment are inconclusive in previous studies, with more studies reporting higher prevalence in females [9,23]. Some studies report a higher prevalence of vascular dementia in men, while females have more Alzheimer’s disease than men [9,10].However, gender differences in the prevalence of vascular dementia reverse with advancing age, with a higher prevalence among women after age 80 [16,24]. Possible reasons for gender differences in the prevalence of cognitive impairment after age 75 identified in our study might be the lifetime accumulation of socioeconomic disadvantages, lack of educational opportunities, and inability of elderly rural females to access an independent income. The poorer socioeconomic status of females identified in our study also supports this view.

Illiteracy was found to have a significant effect on the development of cognitive impairment in females rather than males in our study. This is consistent with previous findings that formal education exerts a protective factor for cognitive impairment and low education is a strong risk factor for Alzheimer’s disease [6,25]. The illiterate women in our study were found to have the highest prevalence of cognitive impairment at 74.1%. Our study also reveals that completing six years of primary school education could protect females against cognitive impairment later in life. The early life of our participants occurred under a traditional feudal society, with a lack of formal education and employment opportunities, and limited exposure to cognitively stimulating environments, such as social engagements, due to the restricted traditional family role of women in rural areas. Less leisure activities and intellectual stimulation throughout their life course may have resulted in limited opportunities for females to build cognitive reserves [17,26]. Our findings are in line with previous studies that report significant interaction between education and economic level, employment, APOE genotype, leisure activity, and social activity on cognitive function, and suggest that education may be a proxy for third variables influencing cognitive function [25,27]. The implementation of nine-year compulsory education system since 1986 has achieved significant progress in eradicating illiteracy in China, however, the gap between urban and rural areas in education quality still remains [28,29]. This calls for measures to be taken to ensure uniform education and to narrow the urban-rural gap. Future studies might follow current middle-aged men and women with better education and employment opportunities, to explore the influence of education on gender differences in cognitive impairment.

Habitual tea consumption was identified in our study to be a protective factor for cognitive impairment in males, which is consistent with several studies reporting that tea consumption is linked to better performance on a wide range of cognitive domains and global cognitive function in older people [30,31]. Biological evidence from experimental studies supports the cardio-protective and neuro-protective role of tea [32]. Tea is enriched in tea polyphenols, which can go through the blood brain barrier to exert neuro-protective effects by anti-oxidation, anti-inflammation, and scavenging free radicals and chelating irons, and therefore protect cognitive function. Furthermore, the effect of tea polyphenols on maintaining healthy blood vessels by regulating cell signal transduction and inhibiting the proliferation and migration of vascular smooth muscle cells also helps decrease vascular associated cognitive impairment [30,33]. Moreover, tea consumption is more common among individuals with higher education, better socio-economic status, higher levels of physical or social activities, and active lifestyles, which could also contribute to the neuro-protective effects [32,33]. The notably lower proportion of female tea consumers identified in the present study supports this view and might explain the non-significant association between tea consumption and cognitive impairment in females.

Sensory changes, including hearing loss and visual impairment, were risk factors for cognitive impairment in both females and males after controlling for socio-demographic, lifestyle, psychosocial and other health-related factors. This is in agreement with the findings of a systematic review synthesizing 40 observational studies from 12 countries, which indicated that age-related hearing loss is a possible biomarker and potentially modifiable risk factor for cognitive decline, cognitive impairment, and dementia [34]. The co-occurrence of vision and hearing impairment is more prevalent than expected by chance alone, and comorbid sensory and cognitive impairments are linked to greater levels of negative functional consequences, including depression, ADL dependence, loneliness, and even death [35,36]. Two possible hypothesized relationships may help to understand the sensory-cognition link. One is that sensory and cognitive impairments may share common risk factors such as age, smoking, genetics or vascular factors [36,37]. Alternatively, there might be a causality that sensory deficits could limit the neural resources needed for the optimal performance of cognitive tasks, and loss of sensory input might directly alter brain structure and function [34,36].The sensory-cognition link provides us the potential for correcting sensory loss to prevent cognitive impairment. Hearing and visual impairment screening is currently included in the free annual health examination for the elderly in China, however, relevant health management is not covered by the basic public health services. Further longitudinal studies and randomized controlled trials are required to inform service development and policy making.

Cardiovascular diseases were unrelated to cognitive impairment in females, despite the higher prevalence. However, in the present study, diabetes was significantly linked with cognitive impairment in males, yielding 2.344 odds of cognitive impairment compared to odds for those without diabetes after controlling for other factors. Cardiovascular risk factors for cognitive impairment have been studied extensively in HICs and urban regions, and currently, a consistent and strong association is only found between diabetes in late-life and the subsequent onset of dementia [3,6]. A recent meta-analysis of 2.3 million individuals reported that type 2 diabetes increased the risk for developing dementia by 60%, and for vascular dementia, the risk was greater in women [38]. In the present study, diabetes was not associated with cognitive impairment in women. Alzheimer’s disease is reported to be more prevalent in rural China than in urban areas, but vascular disease shows an opposite trend [16]. Some risk factors affecting women that are associated with Alzheimer’s disease might interfere with the effect of diabetes [10,39].

Our study agreed with previous studies that cognitive impairment is strongly associated with increasing age and ADL dependence [4,40], with more focus on physical ADL dependence in females. The remarkably higher prevalence of chronic conditions and poorer socio-economic status of females compared to males may have also contributed to greater physical ADL dependence in females. Interestingly, satisfactory neighborhood social interactions were found to be inversely associated with cognitive impairment in men. Potential confounders, such as psychological stress, stimulating environment, and causality relationship, might contribute to these findings. It should be mentioned that the confidence interval for social neighborhood interactions was large (1.424–16.583), which might be a result of the small sample size of male participants living in neighborhoods with poor social interactions with others. Further research is needed to determine these associations.

This study has some limitations. First, a causal relationship between risk factors and the development of cognitive impairment could not be ascertained, due to the cross-sectional study design. Second, measurement of cognitive impairment was based on cognitive testing and might be subject to measurement error. However, cognitive impairment was identified in this study using different MMSE cutoff points, based on the educational attainment of participants. Third, participants of this study were recruited from villages located in one province, and the sample size was relatively small compared to the total number of rural elders in China. Thus, findings of this study cannot be generalized to the whole rural population of China. Finally, the prevalence of cognitive impairment might be underestimated, as individuals with life threatening illnesses did not participate in this study. However, the participation rate for both males and females was generally acceptable and the number of excluded individuals with life threatening illness was small. Future longitudinal studies with larger sample sizes are needed to more accurately ascertain the predictors of cognitive impairment in rural populations and to examine other factors not considered in this study, such as depression, early-life factors, stroke, and life events.

## 5. Conclusions

This study revealed a high prevalence of cognitive impairment in male and female elderly people aged 65 years and older living in rural China, with females showing a significantly higher prevalence than males after age 75 years. Rural elders, especially females, are vulnerable to cognitive impairment due to their low socio-economic status and limited health resources. In the present study, factors associated with cognitive impairment in females and males were found to be different, providing evidence for individualized intervention developments targeting different high-risk populations.

This study has several implications for further research and practice. First, given the significant effect of education on cognitive impairment among females, appropriate education programs should be established for rural women as a public health strategy to alleviate gender disparity and to reduce risks for cognitive impairment. Second, standardized cognitive assessment procedures and measurements suitable for rural and less developed areas which take low education levels and sensory impairment into account should be established to enable the early detection and early prevention of cognitive impairment. Third, considering the multifactorial etiology of cognitive impairment, multidisciplinary collaboration and the integration of more representative technologies are imperative to provide multi-domain interventions for dementia prevention and management.

## Figures and Tables

**Table 1:** Major characteristics of study population.

Characteristics		Male (*n* = 550), n (%)	Female (*n* = 700), *n* (%)	*p*
Demographics				
Age (years)	65~	197(35.8)	236(33.7)	0.744
	70~	175(31.8)	218(31.1)	
	75~	108(19.6)	153(21.9)	
	80~	70(12.7)	93(13.3)	
Marital status	Married	444(80.7)	417(59.6)	0.000
	Divorced/Widowed	106(19.3)	283(40.4)	
Education	Illiteracy	27(4.9)	108(15.4)	0.000
	Primary school	222(40.4)	392(56.0)	
	≥ Secondary school	301(54.7)	200(28.6)	
Income	Subsidies ^§^	219(39.8)	412(58.9)	0.000
	Poverty	115(20.9)	145(20.7)	
	Non-poverty	216(39.3)	143(20.4)	
Living situation	Alone	46(8.4)	93(13.3)	0.000
	With spouse	243(44.2)	212(30.3)	
	2 generations	33(6.0)	61(8.7)	
	≥3 generations	228(41.5)	334(47.7)	
**Lifestyle**				
Regular house/farm work	No	227(41.3)	137(19.6)	0.000
	Yes	323(58.7)	563(80.4)	
Smoking	Never	290(52.7)	693(99.0)	0.000
	Ex-smoker	33(6.0)	6(0.9)	
	Smoker	227(41.3)	1(0.1)	
Alcohol consumption	Never	449(81.6)	699(99.9)	0.000
	Sometimes	79(14.4)	1(0.1)	
	Often	22(4.0)	0(0.0)	
Tea consumption	No	386(70.2)	659(94.1)	0.000
	Yes	164(29.8)	41(5.9)	
**Psychosocial factors**				
Sleep quality	Good	391(71.1)	400(57.1)	0.000
	Moderate	103(18.7)	203(29.0)	
	Poor	56(10.2)	97(13.9)	
Memory complaint	No	120(21.8)	96(13.7)	0.000
	Yes	430(78.2)	604(86.3)	
Social interactions in	Good	172(31.3)	239(34.1)	0.513
neighborhood	Satisfactory	361(65.6)	443(63.3)	
	Poor	17(3.1)	18(2.6)	
Coping	Negative	50(9.1)	75(10.7)	0.342
	Positive	500(90.9)	625(89.3)	

Notes: ^§^ basic living allowance provided by the government for rural elderly, RMB 80 Yuan/month (around 11.5 US dollars).

**Table 2:** Health-related characteristics of study population.

Characteristics		Male (*n* = 550), *n* (%)	Female (*n* = 700), *n* (%)	*p*
BMI (kg/m^2^)	Underweight	44 (8.0)	46 (6.6)	0.188
	Normal	277 (50.4)	328 (46.9)	
	Overweight	229 (41.6)	326 (46.6)	
Waistline (cm)	Normal	265 (48.2)	244 (34.9)	0.000
	High	285 (51.8)	456 (65.1)	
Hearing	Poor	171 (31.1)	223 (31.9)	0.780
	Moderate	219 (39.8)	286 (40.9)	
	Good	160 (29.1)	191 (27.3)	
Vision	Normal	510 (92.7)	635 (90.7)	0.203
	Weak	40 (7.3)	65 (9.3)	
Chronic disease	0	151 (27.5)	139 (19.9)	0.005
	1	204 (37.1)	260 (37.1)	
	2	130 (23.6)	188 (26.9)	
	≥3	65 (11.8)	113 (16.1)	
Hypertension	No	302 (54.9)	335 (47.9)	0.013
	Yes	248 (45.1)	365 (52.1)	
Diabetes	No	516 (93.8)	629 (89.9)	0.012
	Yes	34 (6.2)	71 (10.1)	
Cerebrovascular disease	No	501 (91.1)	655 (93.6)	0.099
	Yes	49 (8.9)	45 (6.4)	
Heart disease	No	407 (74.0)	516 (73.7)	0.909
	Yes	143 (26.0)	184 (26.3)	
Hyperlipidemia	No	522 (94.9)	588 (84.0)	0.000
	Yes	28 (5.1)	112 (16.0)	
Renal disease	No	521 (94.7)	682 (97.4)	0.013
	Yes	29 (5.3)	18 (2.6)	
Traumatic brain injury	No	496 (90.2)	657 (93.9)	0.016
	Yes	54 (9.8)	43 (6.1)	
ADL dependence	No	515 (93.6)	646 (92.3)	0.357
	Yes	35 (6.4)	54 (7.7)	
Physical ADL dependence	No	304 (55.3)	241 (34.4)	0.000
	Yes	246 (44.7)	459 (65.6)	
IADL dependence	No	392 (71.3)	449 (64.1)	0.008
	Yes	158 (28.7)	251 (35.9)	

Notes: BMI, Body Mass Index; ADL, Activities of Daily Living; IADL, instrumental ADL.

**Table 3:** Subgroup analysis of cognitive impairment among males and females.

Subgroup	Male (*n* = 550)		Female (*n* = 700)		*p*
	Individuals, *N*	Cases, *n* (%)	Individuals, *N*	Cases, *n* (%)	
Overall	550	220 (40.0)	700	316 (45.1)	0.068
Age(years)					
65~	197	70 (35.5)	236	70 (29.7)	0.193
70~	175	61 (34.9)	218	84 (38.5)	0.453
75~	108	49 (45.4)	153	96 (62.7)	0.005
80~	70	40 (57.1)	93	66 (71.0)	0.067
Education					
Illiteracy	27	13 (48.1)	108	80 (74.1)	0.009
Primary school	222	66 (29.7)	392	139 (35.5)	0.148
≥Secondary school	301	141 (46.8)	200	97 (48.5)	0.716
Marital status					
Married	444	171 (38.5)	417	160 (38.4)	0.965
Divorced/Widowed	106	49 (46.2)	283	156 (55.1)	0.118
Income					
Subsidies ^§^	219	100 (45.7)	412	209 (50.7)	0.226
Poverty	115	39 (33.9)	145	51 (35.2)	0.832
Non-poverty	216	81 (37.5)	143	56 (39.2)	0.751
Living alone					
Yes	46	19 (41.3)	93	51 (54.8)	0.133
No	504	201 (39.9)	607	265 (43.7)	0.204

Notes: ^§^ basic living allowance provided by the government for rural elderly, RMB 80 Yuan/month (around 11.5 US dollars).

**Table 4:** Prevalence of cognitive impairment and univariate analysis.

Variables		Male (*n* = 550)		Female (*n* = 700)	
		Prevalence (95%CI)	OR (95%CI)	Prevalence (95%CI)	OR (95%CI)
Total		40.0 (35.9–44.1)	-	45.1 (41.4–48.8)	-
Age (years)	65~ (ref.)	35.5 (28.8–42.3)		29.7 (23.8–35.5)	
	70~	34.9 (27.7–42.0)	0.971 (0.634–1.487)	38.5 (32.0–45.0)	1.487 (1.006–2.197)
	75~	45.4 (35.8–54.9)	1.507 (0.934–2.431)	62.7 (55.0–70.5)	3.994 (2.597–6.144)
	80~	57.1 (45.3–60.0)	2.419 (1.387–4.218)	71.0 (61.6–80.4)	5.797 (3.420–9.826)
Marital status	Married (ref.)	38.5 (34.0–43.1)		38.4 (33.7–43.1)	
	Divorced/Widowed	46.2 (36.6–55.9)	1.372 (0.895–2.103)	55.1 (49.3–61.0)	1.973 (1.452–2.680)
Education	Illiteracy (ref.)	48.1 (28.0–68.3)		74.1 (65.7–82.5)	
	Primary school	29.7 (23.7–35.8)	0.456 (0.203–1.022)	35.5 (30.7–40.2)	0.192 (0.119–0.310)
	≥ Secondary school	46.8 (41.2–52.5)	0.949 (0.432–2.087)	48.5 (41.5–55.5)	0.330 (0.198–0.550)
Income	Subsidies^§^(ref.)	45.7 (39.0–52.3)		50.7 (45.9–55.6)	
	Poverty	33.9 (25.1–42.7)	0.611 (0.382–0.976)	35.2 (27.3–43.0)	0.527 (0.356–0.780)
	Non-poverty	37.5 (31.0–44.0)	0.714 (0.487–1.047)	39.2 (31.1–47.3)	0.625 (0.424–0.921)
Regular house/farm work	No (ref.)	48.0 (41.5–54.6)		63.5 (55.3–71.7)	
	Yes	34.4 (29.2–39.6)	0.567 (0.401–0.802)	40.7 (36.6–44.7)	0.394 (0.268–0.580)
Smoking	Never (ref.)	42.8 (37.0–48.5)		45.0 (41.3–48.7)	
	Ex-smoker	42.4 (24.6–60.2)	0.986 (0.476–2.044)	66.7 (12.5–120.9)	2.442 (0.444–13.422)
	Smoker	36.1 (29.8–42.4)	0.757 (0.530–1.082)	0.0	0.000
Alcohol consumption	Never (ref.)	42.8 (38.2–47.4)		45.2 (41.5–48.9)	
	Sometimes	27.8 (17.7–38.0)	0.517 (0.305–0.874)	0.0	0.000
	Often	27.3 (7.1–47.5)	0.502 (0.193–1.307)	-	-
Tea consumption	No (ref.)	43.3 (38.3–48.2)		45.4 (41.6–49.2)	
	Yes	32.3 (25.1–39.6)	0.626 (0.426–0.919)	41.5 (25.7–57.2)	0.853 (0.450–1.617)
Sleep quality	Good (ref.)	39.6 (34.8–44.5)		42.3 (37.4–47.1)	
	Moderate	41.7 (32.1–51.4)	1.091 (0.702–1.696)	50.7 (43.8–57.7)	1.408 (1.003–1.976)
	Poor	39.3 (26.1–52.5)	0.985 (0.555–1.748)	45.4 (35.3–55.4)	1.135 (0.726–1.773)
Memory complaint	No (ref.)	35.8 (27.1–44.5)		29.2 (19.9–38.4)	
	Yes	41.2 (36.5–45.8)	1.253 (0.823–1.906)	47.7 (43.7–51.7)	2.213 (1.386–3.535)
Living situation	Alone (ref.)	41.3 (26.5–56.1)		54.8 (44.5–65.1)	
	With spouse	37.9 (31.7–44.0)	0.866 (0.456–1.645)	38.2 (31.6–44.8)	0.509 (0.311–0.834)
	2 generations	24.2 (8.8–39.7)	0.455 (0.169–1.223)	44.3 (31.4–57.1)	0.654 (0.342–1.252)
	≥3 generations	44.3 (37.8–50.8)	1.130 (0.594–2.149)	47.0 (41.6–52.4)	0.730 (0.460–1.159)
Social interactions in neighborhood	Good (ref.)	40.1 (32.7–47.5)		43.9 (37.6–50.3)	
	Satisfactory	38.2 (33.2–43.3)	0.924 (0.637–1.339)	45.6 (40.9–50.3)	1.070 (0.779–1.468)
	Poor	76.5 (54.0–99.0)	4.851 (1.519–15.497)	50.0 (24.4–75.6)	1.276 (0.489–3.328)
Coping	Negative (ref.)	48.0 (33.7–62.3)		58.7 (47.3–70.1)	
	Positive	39.2 (34.9–43.5)	0.698 (0.390–1.251)	43.5 (39.6–47.4)	0.543 (0.334–0.883)

Notes: ^§^basic living allowance provided by the government for rural elderly, RMB 80 Yuan/month (around 11.5 US dollars).

**Table 5:** Prevalence of cognitive impairment and univariate analysis of health-related factors.

Variables		Male (*n* = 550)		Female (*n* = 700)	
		Prevalence (95%CI)	OR (95%CI)	Prevalence (95%CI)	OR (95%CI)
BMI (kg/m^2^)	Underweight	50.0 (34.6–65.4)		60.9 (46.2–75.5)	
	Normal	40.1 (34.3–45.9)	0.669 (0.353–1.266)	45.7 (40.3–51.2)	0.542 (0.288–1.018)
	Overweight	38.0 (31.7–44.3)	0.613 (0.320–1.172)	42.3 (36.9–47.7)	0.472 (0.251–0.887)
Waistline (cm)	Normal	40.8 (34.8–46.7)		46.3 (40.0–52.6)	
	High	39.3 (33.6–45.0)	0.941 (0.669–1.324)	44.5 (39.9–49.1)	0.930 (0.681–1.271)
Hearing	Poor (ref.)	36.3 (28.7–43.8)		34.6 (27.7–41.4)	
	Moderate	31.5 (25.3–37.7)	0.477 (0.307–0.741)	38.8 (33.1–44.5)	0.319 (0.213–0.477)
	Good	54.4 (46.8–61.9)	0.386 (0.255–0.584)	62.3 (55.9–68.7)	0.383 (0.267–0.550)
Vision	Normal	38.4 (34.2–42.7)		42.2 (38.4–46.1)	
	Weak	60.0 (44.1–75.9)	2.403 (1.245–4.637)	73.8 (62.9–84.8)	3.867 (2.175–6.872)
Chronic disease	0 (ref.)	35.1 (27.4–42.8)		36.0 (27.9–44.0)	
	1	40.7 (33.9–47.5)	1.268 (0.821–1.961)	48.1 (42.0–54.2)	1.648 (1.079–2.517)
	2	39.2 (30.7–47.7)	1.194 (0.735–1.940)	47.3 (40.1–54.5)	1.600 (1.021–2.508)
	≥ 3	50.8 (38.3–63.3)	1.907 (1.057–3.440)	46.0 (36.7–55.3)	1.517 (0.914–2.519)
Hypertension	No (ref.)	39.4 (33.9–44.9)		41.8 (36.5–47.1)	
	Yes	40.7 (34.6–46.9)	1.057 (0.750–1.488)	48.2 (43.1–53.4)	1.297 (0.962–1.749)
Diabetes	No (ref.)	39.0 (34.7–43.2)		44.7 (40.8–48.6)	
	Yes	55.9 (38.3–73.5)	1.985 (0.986–3.996)	49.3 (37.4–61.2)	1.204 (0.737–1.968)
Cerebrovascular disease	No (ref.)	38.9 (34.6–43.2)		44.6 (40.8–48.4)	
	Yes	51.0 (36.5–65.5)	1.635 (0.908–2.944)	53.3 (38.2–68.5)	1.421 (0.775–2.603)
Heart disease	No (ref.)	39.6 (34.8–44.3)		43.8 (39.5–48.1)	
	Yes	41.3 (33.1–49.4)	1.073 (0.728–1.581)	48.9 (41.6–56.2)	1.229 (0.877–1.721)
Hyperlipidemia	No (ref.)	40.0 (35.8–44.3)		46.3 (42.2–50.3)	
	Yes	39.3 (20.0–58.6)	0.969 (0.445–2.110)	39.3 (30.1–48.5)	0.752 (0.498–1.135)
Renal disease	No (ref.)	39.9 (35.7–44.1)		44.7 (41.0–48.5)	
	Yes	41.4 (22.3–60.4)	1.062 (0.497–2.270)	61.1 (36.2–86.1)	1.942 (0.744–5.071)
Traumatic brain injury	No (ref.)	38.7 (34.4–43.0)		44.4 (40.6–48.3)	
	Yes	51.9 (38.1–65.6)	1.705 (0.971–2.996)	55.8 (40.3–71.3)	1.579 (0.848–2.939)
ADL dependence	No (ref.)	38.1 (33.9–42.3)		42.7 (38.9–46.5)	
	Yes	68.6 (52.4–84.8)	3.551 (1.702–7.409)	74.1 (62.0–86.1)	3.830 (2.043–7.179)
Physical ADL dependence	No (ref.)	34.5 (29.2–39.9)		33.2 (27.2–39.2)	
	Yes	46.7 (40.5–53.0)	1.664 (1.179–2.348)	51.4 (46.8–56.0)	2.130 (1.539–2.947)
IADL dependence	No (ref.)	35.7 (31.0–40.5)		39.4 (34.9–44.0)	
	Yes	50.6 (42.8–58.5)	1.846 (1.270–2.684)	55.4 (49.2–61.6)	1.907 (1.395–2.607)

Notes: BMI, Body Mass Index; ADL, Activities of Daily Living; IADL, instrumental ADL.

**Table 6:** Multivariate analysis of factors associated with cognitive impairment.

Variables		Male (*n*-550)	Female (*n* = 700)
		OR (95% CI)	OR (95% CI)
Age (years)	65~(ref.)		
	70~	0.894 (0.559–1.430)	1.133 (0.739–1.736)
	75~	1.179 (0.690–2.014)	2.793 (1.729–4.511)
	80~	2.357 (1.255–4.428)	2.070 (1.061–4.036)
Education	Illiteracy (ref.)		
	Primary school	0.754 (0.304–1.866)	0.352 (0.199–0.623)
	≥ Secondary school	2.156 (0.868–5.355)	0.851 (0.454–1.597)
Tea consumption	No (ref.)		-
	Yes	0.656 (0.431–0.999)	
Social interactions in neighborhood	Good (ref.)		-
	Satisfactory	0.942 (0.625–1.422)	
	Poor	4.860 (1.424–16.583)	
Hearing	Good (ref.)		
	Moderate	0.830 (0.516–1.334)	0.903 (0.589–1.382)
	Poor	2.027 (1.211–3.393)	2.110 (1.321–3.369)
Vision	Normal (ref.)	-	
	Weak		2.024 (1.051–3.897)
Diabetes	No (ref.)		
	Yes	2.344 (1.083–5.076)	-
ADL dependence	No (ref.)		
	Yes	3.267 (1.459–7.314)	2.041 (0.976–4.270)
Physical ADL dependence	No (ref.)	-	
	Yes		1.613 (1.121–2.322)

Notes: ADL, Activities of Daily Living.
